# FRAX score predicts vertebral compression fractures impairing spinal alignment and hip function after total hip arthroplasty: a retrospective cohort study

**DOI:** 10.1186/s42836-025-00345-8

**Published:** 2025-12-08

**Authors:** Masashi Shimoda, Hyonmin Choe, Hiroyuki Ike, Hideo Mitsui, Koki Abe, Yuta Hieda, Naomi Kobayashi, Yutaka Inaba

**Affiliations:** 1 Department of Orthopedic Surgery, Yokohama City University, 3-9 Fukuura, Kanazawa-Ku, Yokohama, Kanagawa 236-0004 Japan; 2 Department of Orthopedic Surgery, Yokohama City University Medical Center, 4-57 Urafune-cho, Minami-Ku, Yokohama, Kanagawa 232-0024 Japan

**Keywords:** Total hip arthroplasty, Vertebral compression fractures, Standing sagittal spinal alignment, Fracture Risk Assessment Tool score

## Abstract

**Background:**

Vertebral compression fractures (VCFs) can impair posture, gait, and daily activities in patients undergoing total hip arthroplasty (THA). However, limited data are available regarding the incidence, risk factors, and impact of VCFs on sagittal spinal alignment following THA. Therefore, the purpose of this study was to investigate the incidence and risk factors of VCFs after THA, and to evaluate their impact on sagittal spinal alignment and clinical outcomes.

**Methods:**

This retrospective cohort study included 220 patients (243 hips) who underwent primary THA, with a mean follow-up period of 6.1 years. Data collected included patient demographics, Fracture Risk Assessment Tool (FRAX) scores, lumbar bone mineral density measured before THA, sagittal spinal alignment parameters, Harris Hip Score (HHS), and the occurrence of new VCFs. We analyzed changes in spinal alignment and identified risk factors associated with incident VCFs.

**Results:**

VCFs occurred in 20% of hips during the follow-up period. Patients who developed VCFs demonstrated a significantly increased sagittal vertical axis, reduced lumbar lordosis angle, and lower postoperative HHS compared to those without VCFs. Preexisting VCF and higher preoperative FRAX scores were significantly associated with the development of new VCFs. Multivariate logistic regression analysis identified the FRAX score as an independent predictor of incident VCFs.

**Conclusions:**

In this 6.1-year retrospective cohort study, 20% of hips developed new VCFs after THA, which were associated with worsened spinal alignment and hip function. The higher FRAX score, calculated prior to THA surgery, is a useful predictor of VCF risk and may help identify individuals who require closer monitoring or preventive interventions during follow-up after surgery.

Video Abstract

**Supplementary Information:**

The online version contains supplementary material available at 10.1186/s42836-025-00345-8.

## Introduction

Vertebral compression fractures (VCFs) are fragility fractures that increase in frequency with age. VCFs often occur without trauma and may progress with minimal symptoms; however, they are associated with pain, postural deformities, reduced physical function, and increased mortality [1]. The prevalence of VCFs increases with age, affecting up to 45% of individuals in their 70s [2–4]. Because VCFs can impair spinal and pelvic alignment [1], they may have significant implications for patients undergoing lower extremity arthroplasty. However, the specific impact of VCFs on spinopelvic balance following total hip arthroplasty (THA) has not been fully elucidated.

THA is a commonly performed reconstructive procedure for osteoarthritis and osteonecrosis of the femoral head, typically indicated in patients in their 60s and 70s [5, 6]. In this population, both preexisting and newly developed lumbar VCFs warrant careful consideration, as pelvic retroversion secondary to VCFs has been associated with an increased risk of postoperative dislocation, a serious complication following THA [7, 8]. Thus, patients undergoing THA are at increased risk of incident VCFs, which may influence postoperative spinopelvic sagittal alignment. However, limited research has clearly reported the incidence, risk factors, and consequences of VCFs in this patient population. We hypothesized that THA patients with skeletal fragility (e.g., low BMD, high FRAX scores, or preexisting VCFs) would demonstrate increased rates of postoperative VCFs and associated spinopelvic malalignment.

This study aimed to determine the incidence of VCFs during long-term follow-up exceeding five years after THA, evaluate the impact of VCFs on standing sagittal spinal alignment, and identify patient-related factors associated with VCF development.

## Materials and methods

### Ethics approval and informed consent statement

This retrospective study was approved by the institutional ethics review board of Yokohama City University (approval number: F240400049). All methods were performed in accordance with the relevant guidelines and regulations. Informed consent was waived by the Institutional Review Board of Yokohama City University due to the retrospective nature of the study. Participants were allowed to opt out of participation through public notification.

### Participants

**Fig. 1:**
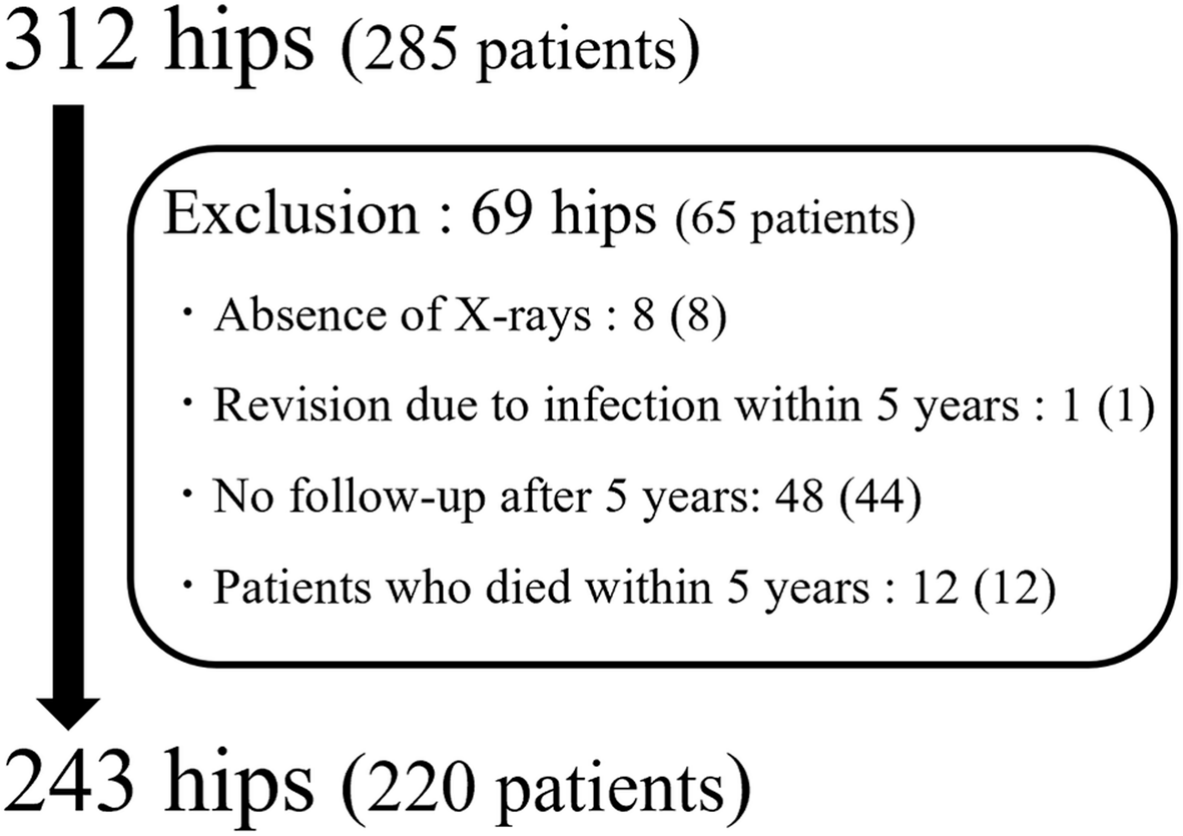
Study participants. Of the 285 patients (312 hips) who underwent THA at our hospital between January 2016 and December 2018, 220 patients (243 hips) were included. Exclusions were 8 hips lacking preoperative and 5-year postoperative radiographs, 48 hips without postoperative follow-up, 12 hips of patients whose death occurred within 5 years postoperatively, and one hip undergoing revision within 5 years postoperatively

In this retrospective study, records of 312 hips from 285 consecutive patients who underwent primary THA at our hospital between January 2016 and December 2018 were reviewed. No age restriction was applied as an eligibility criterion, as we aimed to evaluate vertebral compression fractures across the full spectrum of patients undergoing THA. Exclusion criteria included failure to follow up for over 5 years, revision due to infection within 5 years, and death within 5 years. After exclusions, the study cohort comprised 243 hips from 220 patients (Fig. 1). Analyses were performed per hip. In cases where bilateral THA was performed on the same day, the procedure was counted as one hip to avoid duplication.

### Patients’ backgrounds and postoperative measurements

Patient data included age, sex, body mass index (BMI), preoperative blood test results, preoperative lumbar bone mineral density (BMD) (density and T-score), Fracture Risk Assessment Tool (FRAX) score, preexisting VCFs, Harris Hip Score (HHS) [10], and preoperative radiographs of standing sagittal spinal and pelvic alignments. All patients underwent preoperative BMD measurement and standing spinal radiography as part of the standard evaluation. At the 5- to 7-year follow-up, incident VCFs, standing sagittal spinal radiographs, and HHS were evaluated. The HHS was chosen because it effectively assesses hip function and is highly relevant for evaluating clinical outcomes after THA [9, 10].

Osteoporosis treatment included bisphosphonates (alendronate, risedronate, ibandronate, zoledronate, minodronate), denosumab, teriparatide, romosozumab, selective estrogen receptor modulators, and vitamin D analogs, administered either preoperatively or postoperatively. Information regarding adherence to treatment and follow-up reassessment with DEXA or FRAX was not consistently available.

Preoperative blood tests assessed total protein, albumin, Alkaline phosphatase (ALP), and calcium levels. Sagittal spinal alignment was measured using standing radiographs based on the Jackson method [11]. The C7 sagittal vertical axis was defined as the distance between the C7 plumb line and the posterior superior margin of the sacrum, with a positive value indicating anterior displacement and a negative value indicating posterior displacement. Lumbar lordosis angle was measured as the angle between the superior margins of L1 and S1. Sacral slope angle was defined as the angle between the sacral endplate and the horizontal plane. Pelvic tilt angle was defined as the angle between the midpoint of both femoral heads and the midpoint of the superior margin of S1.

BMD was assessed using a horizontal X-ray bone densitometer (Hologic, Marlborough, MA, USA), and the FRAX score was calculated based on preoperative femoral neck bone density data [12].

VCFs were identified using a quantitative measurement method based on lateral radiographic examinations of the thoracolumbar spine. A VCF was defined when any of the following criteria were met: a central-to-anterior (C/A) or central-to-posterior (C/P) height ratio of less than 0.8, an anterior-to-posterior (A/P) height ratio of less than 0.75, or a reduction of ≥ 20% in vertebral body height (anterior, central, or posterior) compared with adjacent vertebrae. In cases of uniformly flattened vertebrae, a height reduction of ≥ 20% from both adjacent vertebrae was also considered indicative of VCF [13, 14]. These criteria are based on a quantitative morphometric method using thresholds consistent with those proposed by Genant et al. [13], which have been widely adopted for the clinical evaluation of osteoporotic vertebral fractures.

The incidence and distribution of VCFs were evaluated during the follow-up period. Incident VCF was defined as a new fracture identified on follow-up radiographs that was not present on preoperative radiographs. The incidence was calculated as the number of hips with incident VCFs divided by the total number of hips included in the study cohort.

### Effect of VCF on standing posture, clinical outcome, and risk factors for VCF

Radiographic examinations of the thoracolumbar spine were used to assess sagittal spinal alignment parameters. Patients were routinely followed at 3 months, 1 year, and annually thereafter after THA. For the present analysis, preoperative radiographs were obtained at the outpatient clinic before THA surgery, and postoperative radiographs for sagittal alignment assessment were obtained at the 5-year follow-up visit. Hips were first classified based on preoperative radiographs into those with preexisting vertebral compression fractures (With Preexisting VCFs) and those without preexisting vertebral compression fractures (Without Preexisting VCFs). Subsequently, based on radiographic findings during the 5-year postoperative follow-up, patients were grouped into those with incident vertebral compression fractures (With Incident VCFs) and those without incident vertebral compression fractures (Without Incident VCFs). Comparative analyses were performed between these groups, and the association between preoperative patient factors and the development of postoperative VCFs was also investigated.

Comparisons between these groups were conducted as follows: (1) With vs. Without Preexisting VCFs were compared for demographics, preoperative spinal alignment, and clinical outcomes; (2) With vs. Without Incident VCFs were compared for demographics, biochemical markers, spinal alignment changes, and Harris Hip Scores (HHS); and (3) preoperative factors such as FRAX score, BMD, and preexisting VCFs were analyzed for their association with the development of incident VCFs using univariate and multivariate logistic regression.

### Data analysis

Statistical analyses were performed using SPSS software, version 21.0 (IBM Corp., Armonk, NY, USA). The Student’s t-test and the chi-square test were used to compare continuous and categorical variables, respectively. For continuous data that were not normally distributed or exhibited unequal variances, the nonparametric Mann–Whitney U test was applied.

Multivariable logistic regression analysis was performed to identify independent predictors of incident VCF. Given the limited number of events, we restricted the number of covariates according to the rule of at least 10 events per variable to avoid overfitting. Candidate predictors were selected based on clinical relevance and univariate analysis (*P* < 0.20). Specifically, the independent variables included FRAX score, preexisting VCFs, BMI, and preoperative pelvic tilt angle. To avoid multicollinearity, variables already incorporated in the FRAX score (e.g., age, prior fracture, BMD) were not included simultaneously with FRAX. A *P*-value of < 0.05 was considered statistically significant.

## Results

### Patient characteristics

A total of 243 hips were included in this study, comprising 64 hips from men and 179 hips from women. The mean age at surgery was 61.7 years (range, 24–89). The primary diagnoses were osteoarthritis in 193 hips (79%), osteonecrosis of the femoral head in 38 hips (16%), and other conditions in 12 hips (5%), including rheumatoid arthritis in 6 hips, rapid destructive coxopathy in 3 hips, pigmented villonodular synovitis in 1 hip, femoral neck fracture in 1 hip, and proliferative osteochondropathy in 1 hip (Table 1).

**Table 1:** Patient’s background data

Parameters	
Age	61.7 years (range, 24–89)
Sex	
Women	179 hips (74%)
Men	64 hips (26%)
Primary diagnosis	
Osteoarthritis	193 hips (79%)
Osteonecrosis of the femoral head	38 hips (16%)
Other conditions	12 hips (5%)
*T*-score (lumbar spine)	
< −2.5	86 hips (35%)
−1.0 to ≤ −2.5	89 hips (37%)
History of vertebral compression fracture	46 hips (19%)
Osteoporosis treatment	
Before surgery	47 hips (19%)
Initiated during postoperative period	89 hips (37%)

Preoperative BMD assessment revealed that 86 hips (35%) had T-scores below −2.5, and 89 hips (37%) had T-scores between −1.0 and −2.5. Among these, 47 hips (19%) received osteoporosis treatment before surgery, while 89 hips (37%) received treatment postoperatively.

### Comparison between patients with and without preexisting VCFs

**Fig. 2:**
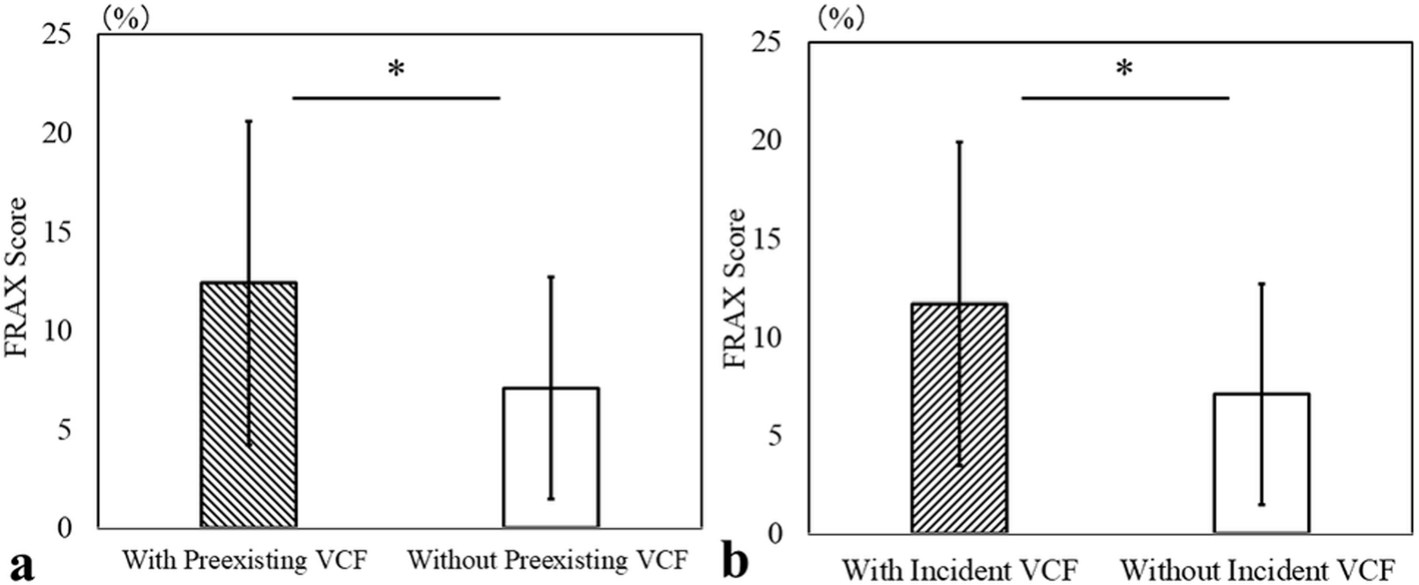
Comparison of FRAX scores between groups. (a) Comparison of FRAX scores between patients with and without preexisting VCFs. Patients with preexisting VCFs had significantly higher FRAX scores than those without (12.4 ± 8.2% vs. 7.1 ± 5.6%, *P* < 0.001). (b) Comparison of FRAX scores between patients with and without incident VCFs. Patients who developed incident VCFs showed significantly higher FRAX scores compared with those without (11.7 ± 9.0% vs. 7.1 ± 5.3%, *P* < 0.001). An asterisk indicates a statistically significant difference (*P* < 0.05)

Among the 243 hips, 46 (19%) had preexisting VCFs. Hips with preexisting VCFs were significantly older (66.4 ± 10.5 vs. 60.6 ± 12.5 years, *P* = 0.009), had higher ALP levels (285.1 ± 94.6 vs. 253.1 ± 81.6 U/L, *P* = 0.024), and higher FRAX scores (12.4 ± 8.2% vs. 7.1 ± 5.6%, *P* < 0.001) (Fig. 2a) compared with those without preexisting VCFs. No significant differences were found in lumbar BMD (0.9 ± 0.2 vs. 1.0 ± 0.2 g/cm^2^, *P* = 0.520) or T-score (−0.7 ± 1.7 vs. −0.5 ± 1.9, *P* = 0.445) (Table 2).

**Table 2:** Comparison of data between hips that had and who that did not have preoperative vertebral compression fracture

	**With Preexisting VCFs**	**Without Preexisting VCFs**	***P*-value**
Age at surgery (years)	66.4 ± 10.5	60.6 ± 12.5	0.009*
BMI (kg/m^2^)	23.5 ± 3.3	23.4 ± 4.1	0.398
Lumbar BMD (g/cm^2^)	0.9 ± 0.2	1.0 ± 0.2	0.520
Lumbar spine T-score	−0.7 ± 1.7	−0.5 ± 1.9	0.445
FRAX score (%)	12.4 ± 8.2	7.1 ± 5.6	< 0.001*
TP (g/dL)	7.1 ± 0.4	7.2 ± 0.4	0.330
Alb (g/dL)	4.2 ± 0.3	4.3 ± 0.4	0.177
ALP (U/L)	285.1 ± 94.6	253.2 ± 81.6	0.024*
Ca (mg/dL)	9.4 ± 0.4	9.4 ± 0.4	0.481
Preoperative SVA (mm)	52.7 ± 51.6	26.0 ± 39.9	0.002*
Preoperative LL (°)	41.9 ± 14.6	48.7 ± 11.6	0.004*
Preoperative SS (°)	36.0 ± 9.5	38.8 ± 8.0	0.072
Preoperative PT (°)	15.6 ± 10.3	13.1 ± 10.3	0.106

^*^P < 0.05, nonparametric Mann–Whitney U test

With Preexisting VCFs, patients who had preoperative vertebral compression fracture; Without Preexisting VCFs, patients who did not have preoperative vertebral compression fracture; BMI, body mass index; BMD, bone mineral density; FRAX, Fracture Risk Assessment Tool; TP, total protein; Alb, albumin; ALP, alkaline phosphatase; Ca, calcium; SVA, sagittal vertical axis; LL, lumbar lordosis angle; SS, sacral slope angle; PT, pelvic tilt angle

Preoperative standing sagittal spinal alignment in the patients with preexisting VCF group showed the following values: sagittal vertical axis, 52.7 ± 51.6 mm; lumbar lordosis angle, 41.9 ± 14.6°; sacral slope angle, 36.0 ± 9.5°; and pelvic tilt angle, 15.6 ± 10.3°. In contrast, patients without preexisting VCFs had a sagittal vertical axis of 26.0 ± 39.9 mm, lumbar lordosis angle of 48.7 ± 11.6°, sacral slope angle of 38.8 ± 8.0°, and pelvic tilt angle of 13.1 ± 10.3°. The hips with preexisting VCF group showed significantly greater sagittal vertical axis (*P* = 0.002) and lower lumbar lordosis angle (*P* = 0.004) (Table 2).

### Incidence and distribution of incident VCFs

The mean follow-up period was 6.1 years (range, 5.0–8.0 years). No artificial joint dislocations or peri-implant fractures occurred during the study period. Incident VCFs were observed in 49 hips (20%). Among these 49 hips, only 12 hips (24%) had received preoperative osteoporosis treatment. The incidence of incident VCFs was significantly higher in hips with preexisting VCFs compared with those without, as determined by a chi-square test (*P* = 0.002). However, neither preoperative nor postoperative osteoporosis treatment was significantly associated with the occurrence of incident VCFs, as determined by chi-square tests (*P* = 0.313 and *P* = 0.596, respectively).

The incident VCFs included 47 single-vertebra fractures (T11, 15; T12, 15; L1, 10; L2, 4; L3, 2; L4, 1) and two cases involving two vertebrae (L1 and L3, 1; T12 and L4, 1), with the thoracolumbar junction (T11–L2) affected in 46 hips (92%).

### Comparison between patients with and without incident VCFs

Significant differences were observed in mean age at surgery (66.6 ± 11.4 vs. 60.5 ± 12.3 years, *P* = 0.01), BMI (24.4 ± 3.7 vs. 23.1 ± 3.9 kg/m^2^, *P* = 0.015), and FRAX score (11.7 ± 9.0% vs. 7.1 ± 5.3%, *P* < 0.001) (Fig. 2b)), indicating elderly and obese patients are more likely to develop incident VCFs, and our findings suggest that a higher FRAX score, which reflects these factors, may be associated with an increased risk of incident VCF. No significant differences were found in lumbar BMD (1.0 ± 0.2 vs. 0.9 ± 0.2 g/cm^2^, *P* = 0.243) or T-score (− 0.4 ± 1.5 vs. −0.6 ± 2.0, *P* = 0.238) (Table 3).

**Table 3:** Comparison of data between hips that had and who that did not have postoperative vertebral compression fracture

	**With NIncident VCFs**	**Without Incident VCFs**	***P*-value**
Age at surgery (years)	66.6 ± 11.4	60.5 ± 12.3	0.01*
BMI (kg/m^2^)	24.4 ± 3.7	23.1 ± 3.9	0.015*
Lumbar BMD(g/cm^2^)	1.0 ± 0.2	1.0 ± 0.2	0.243
Lumbar spine T-score	−0.4 ± 1.5	−0.6 ± 2.0	0.238
FRAX score (%)	11.7 ± 9.0	7.1 ± 5.3	< 0.001*
TP (g/dL)	7.2 ± 0.4	7.1 ± 0.4	0.355
Alb (g/dL)	4.2 ± 0.3	4.3 ± 0.3	0.094
ALP (U/L)	289.2 ± 98.7	251.5 ± 79.4	0.011*
Ca (mg/dL)	9.4 ± 0.3	9.4 ± 0.4	0.804
Preoperative SVA (mm)	40.5 ± 46.8	28.5 ± 42.4	0.078
Preoperative LL (°)	44.5 ± 13.6	48.1 ± 12.1	0.151
Preoperative SS (°)	36.7 ± 9.5	38.6 ± 8.0	0.162
Preoperative PT (°)	17.3 ± 10.6	12.2 ± 9.8	0.007*

^*^P < 0.05, nonparametric Mann–Whitney U test

With Incident VCFs, patients who had postoperative vertebral compression fracture; Without Incident VCFs, patients who did not have postoperative vertebral compression fracture; BMI, body mass index; BMD, bone mineral density; FRAX, Fracture Risk Assessment Tool; TP, total protein; Alb, albumin; ALP, alkaline phosphatase; Ca, calcium; SVA, sagittal vertical axis; LL, lumbar lordosis angle; SS, sacral slope angle; PT, pelvic tilt angle

Preexisting VCFs were significantly associated with incident VCF occurrence (*P* = 0.002), while osteoporosis treatments were not (*P* = 0.313 and *P* = 0.596, respectively). ALP levels were higher in hips with incident VCFs (289.2 ± 98.7 vs. 251.5 ± 79.4 U/L, *P* = 0.011). Pelvic tilt angle was significantly greater in these hips (17.3 ± 10.6° vs. 12.2 ± 9.8°, *P* = 0.007), although no significant differences were found in sagittal vertical axis, lumbar lordosis angle, or sacral slope angle (Table 3).

### Postoperative spinal alignment and clinical outcomes

Hips with preexisting VCFs had significantly higher postoperative sagittal vertical axis (67.0 ± 68.4 mm vs. 39.4 ± 46.0 mm, *P* = 0.036), lower lumbar lordosis angle (35.0 ± 16.6° vs. 44.0 ± 12.8°, *P* < 0.001), lower sacral slope angle (29.7 ± 9.8° vs. 34.8 ± 7.6°, *P* < 0.001), and higher pelvic tilt angle (21.3 ± 10.4° vs. 15.8 ± 10.1°, *P* = 0.003) (Table 4). Changes from preoperative to postoperative alignment were also significantly different in lumbar lordosis angle (−7.0 ± 6.8° vs. −4.7 ± 7.3°, *P* = 0.014), sacral slope angle (−6.2 ± 3.8° vs. −4.0 ± 5.1°, *P* = 0.001), and pelvic tilt angle (5.7 ± 7.4° vs. 2.3 ± 5.9°, *P* = 0.003).

**Table 4:** Comparison of postoperative standing spinal sagittal alignment and Harris hip scores between hips that had and who that did not have preoperative vertebral compression fracture

	**With Preexisting VCFs**	**Without Preexisting VCFs**	***P*-value**
Postoperative SVA (mm)	67.0 ± 68.4	39.4 ± 46.0	0.036*
Postoperative LL (°)	35.0 ± 16.6	44.0 ± 12.8	< 0.001*
Postoperative SS (°)	29.7 ± 9.8	34.8 ± 7.6	< 0.001*
Postoperative PT (°)	21.3 ± 10.4	15.8 ± 10.1	0.003*
ΔSVA (mm)	16.8 ± 48.9	9.8 ± 33.2	0.464
ΔLL (°)	−7.0 ± 6.8	−4.7 ± 7.3	0.014*
ΔSS (°)	−6.2 ± 3.8	−4.0 ± 5.1	0.001*
ΔPT (°)	5.7 ± 7.4	2.3 ± 5.9	0.003*
Preoperative HHS	52.6 ± 15.4	58.0 ± 14.9	0.040*
Postoperative HHS	85.2 ± 12.7	88.9 ± 13.7	0.026*

^*^P < 0.05, nonparametric Mann–Whitney U test

HHS, Harris hip score; With Preexisting VCFs, patients who had preoperative vertebral compression fracture; Without Preexisting VCFs, patients who did not have preoperative vertebral compression fracture; SVA, sagittal vertical axis; LL, lumbar lordosis angle; SS, sacral slope angle; PT, pelvic tilt angle; ΔSVA, preoperative to postoperative change in SVA; ΔLL, preoperative to postoperative change in LL; ΔPT, preoperative to postoperative change in PT; ΔSS, preoperative to postoperative change in SS

**Fig. 3:**
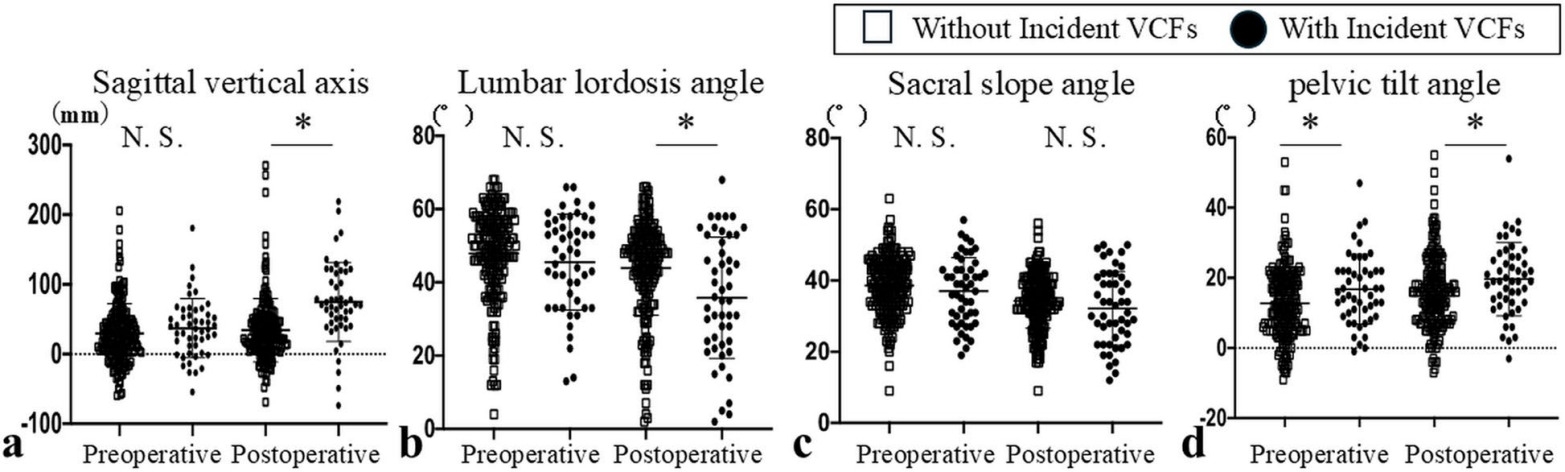
Comparison of preoperative and postoperative standing spinal sagittal plane alignment. (**a)** sagittal vertical axis, (**b**) lumbar lordosis angle, (**c**) sacral slope angle, (**d**) pelvic tilt angle. Patients with incident VCFs demonstrate significant differences in postoperative sagittal vertical axis, lumbar lordosis angle, and pelvic tilt angle compared to those without incident VCFs, with no significant difference in postoperative sacral slope angle (a–d). N.S., not significant. An asterisk indicates a statistically significant difference (*P* < 0.05)

Hips with incident VCFs showed significantly greater postoperative sagittal vertical axis (78.7 ± 62.8 mm vs. 35.2 ± 44.5 mm, *P* < 0.001), lower lumbar lordosis angle (35.1 ± 17.0° vs. 44.1 ± 12.6°, *P* < 0.001), and higher pelvic tilt angle (20.3 ± 11.5° vs. 16.0 ± 9.9°, *P* = 0.010). No significant difference was found in sacral slope angle (31.9 ± 10.4° vs. 34.4 ± 7.6°, *P* = 0.110) (Table 5, Fig. 3). Significant changes from pre- to postoperative alignment were observed in sagittal vertical axis (39.2 ± 36.7 mm vs. 3.8 ± 33.1 mm, *P* < 0.001) and lumbar lordosis angle (−9.8 ± 9.7° vs. −4.0 ± 6.0°, *P* < 0.001) (Table 5).

**Table 5:** Comparison of postoperative standing spinal sagittal alignment and Harris hip scores between hips that had and who that did not have postoperative vertebral compression fracture

	**With Incident VCFs**	**Without Incident VCFs**	***P*-value**
Postoperative SVA (mm)	78.7 ± 62.8	35.2 ± 44.5	< 0.001*
Postoperative LL (°)	35.1 ± 17.0	44.1 ± 12.6	< 0.001*
Postoperative SS (°)	31.9 ± 10.4	34.4 ± 7.6	0.110
Postoperative PT (°)	20.3 ± 11.5	16.0 ± 9.9	0.010*
ΔSVA (mm)	39.2 ± 36.7	3.8 ± 33.1	< 0.001*
ΔLL (°)	−9.8 ± 9.7	−4.0 ± 6.0	< 0.001*
ΔSS (°)	−4.8 ± 4.7	−4.3 ± 5.0	0.586
ΔPT (°)	3.0 ± 6.7	3.3 ± 6.2	0.662
Preoperative HHS	52.8 ± 16.8	58.0 ± 14.5	0.046*
Postoperative HHS	82.2 ± 15.0	89.7 ± 12.8	< 0.001*

^*^P < 0.05, nonparametric Mann–Whitney U test

HHS, Harris hip score; With Incident VCFs, patients who had postoperative vertebral compression fracture; Without Incident VCFs, patients who did not have postoperative vertebral compression fracture; SVA, sagittal vertical axis; LL, lumbar lordosis angle; SS, sacral slope angle; PT, pelvic tilt angle; ΔSVA, preoperative to postoperative change in SVA; ΔLL, preoperative to postoperative change in LL; ΔPT, preoperative to postoperative change in PT; ΔSS, preoperative to postoperative change in SS

In terms of clinical outcomes, patients with preexisting VCFs had significantly lower HHS both preoperatively (52.6 ± 15.4 vs. 58.0 ± 14.9,* P* = 0.040) and postoperatively (85.2 ± 12.7 vs. 88.9 ± 13.7, *P* = 0.026) (Table 4). Similarly, hips with incident VCFs had lower preoperative (52.8 ± 16.8 vs. 58.0 ± 14.5, *P* = 0.046) and postoperative HHS (82.2 ± 15.0 vs. 89.7 ± 12.8, P < 0.001) than those without (Table 5).

### Predictors of postoperative VCF occurrence

**Fig. 4:**
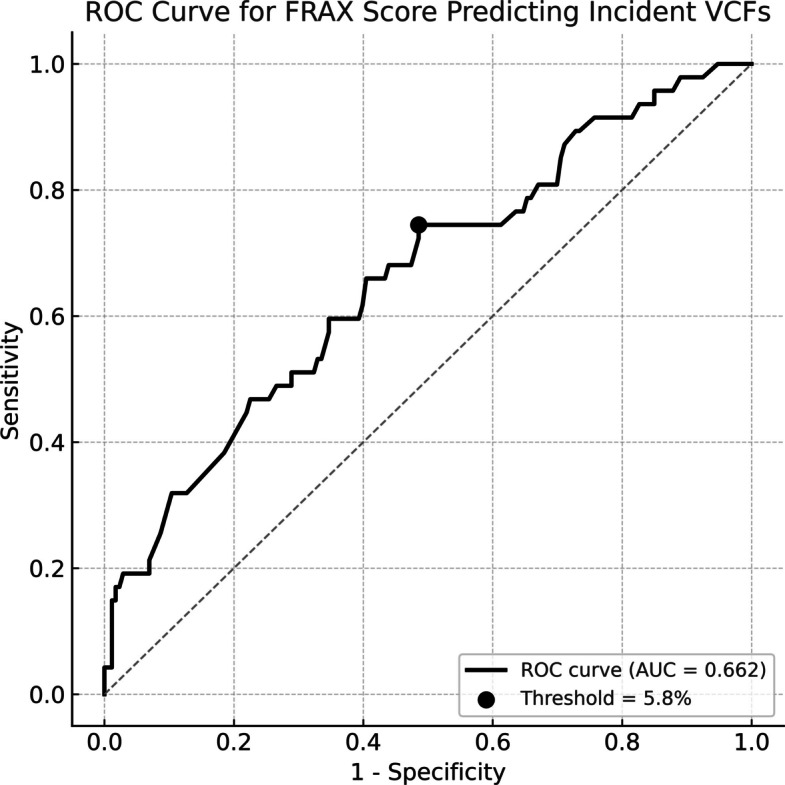
Receiver operating characteristic (ROC) curve of the FRAX score for predicting incident VCFs after THA. The area under the curve (AUC) was 0.662, indicating moderate discriminatory ability. The optimal cut-off value, determined by the Youden index, was 5.8%, which provided a sensitivity of 74% and a specificity of 51%. The black circle marks the cut-off point on the ROC curve

Multivariable logistic regression identified the FRAX score as a significant predictor of incident VCF (Table 6). Receiver operating characteristic (ROC) analysis demonstrated moderate discriminative ability of the FRAX score, with an AUC of 0.66 (Fig. 4). The optimal cutoff determined by the Youden index was 5.8%, yielding a sensitivity of 74% and a specificity of 51%. For clinical interpretation, a cutoff of 10% provided a sensitivity of 47% and a specificity of 77%, while a cutoff of 15% yielded a sensitivity of 32% and a specificity of 90% (Table 7). The odds ratio for incident VCF per one-point increase in the FRAX score was 1.09 (95% CI: 1.03–1.15), indicating that higher FRAX scores were associated with an increased risk of postoperative VCF.

**Table 6:** Results of the multivariate analysis

	**Odds ratio**	**95% Confidence interval**	
		**Lower limit**	**Upper limit**
FRAX score	1.088	1.031	1.147
Preexisting VCFs	1.532	0.667	3.519
Preoperative PT	1.029	0.994	1.064
BMI	1.084	0.997	1.177

FRAX, Fracture Risk Assessment Tool; Preexisting VCFs, patients who had preoperative vertebral compression fractures; PT, pelvic tilt angle; BMI, body mass index; BMD

**Table 7:** Sensitivity and specificity of FRAX score at different cutoff values for predicting incident VCF

FRAX score (%)	Sensitivity (%)	Specificity (%)
5	74	43
10	47	77
15	32	90
20	17	98
25	15	99
30	4	100

FRAX, Fracture Risk Assessment Tool

## Discussion

VCFs are known to affect spinopelvic alignment and increase the risk of hip dislocation following THA [7]. Therefore, understanding the incidence and impact of postoperative VCFs is clinically important. In this study, VCFs developed in 20% of hips during an average follow-up of 6.1 years. Given their associations with spinopelvic imbalance, lumbar kyphosis, decreased functional capacity, and increased mortality, prevention of VCF is essential to reduce postoperative complications in THA.

In THA, monitoring postoperative changes in sagittal alignment—including global spinal alignment, lumbar lordosis angle, and pelvic tilt angle—is critical, as these factors influence implant stability, dislocation risk, and long-term outcomes [15]. In particular, minimizing postoperative increase in pelvic tilt angle is important to reduce the risk of anterior dislocation [8]. However, age-related postural changes are often unavoidable in elderly patients, and VCFs may contribute to these alterations. Previous studies have linked VCFs to increased posterior pelvic tilt angle following THA [7], underscoring the importance of postoperative management of spinopelvic alignment.

VCFs decrease the lumbar lordosis angle, shifting the center of gravity anteriorly (i.e., increasing sagittal vertical axis), which leads to compensatory posterior pelvic tilt angle and knee flexion, ultimately reducing gait function [16]. In our study, hips with incident VCFs showed significantly greater changes in the sagittal vertical axis and lumbar lordosis angle compared to those without VCFs, indicating anterior translation of the C7 plumb line and loss of lumbar lordosis. Moreover, hips with preexisting VCFs exhibited greater changes in lumbar lordosis angle, pelvic tilt angle, and sacral slope angle than those without, suggesting a predisposition toward postoperative lumbar kyphosis and pelvic retroversion. Importantly, hips with either preexisting or incident VCFs had significantly lower HHS at five years postoperatively, indicating that VCF prevention is key to improving satisfaction and functional outcomes after THA. Although no dislocations occurred during our 6-year observation period, previous studies have reported progressive increases in pelvic tilt angle beyond five years. Therefore, long-term monitoring of pelvic tilt angle and dislocation risk is warranted in patients with VCFs [17].

VCF prevalence varies by region and age. In the UK, 20% of women over age 65 are affected [18]; in the US, the prevalence is 16.2% in those aged 65–69 years and 21.9% in those aged 70–74 years [19]. In Hong Kong, it is 13% for those aged 65–69[20]; in Vietnam, 23% of men and 26% of women over 50 have VCFs [21]. In Japan, prevalence ranges from 7.6–14% in those in their 60 s and 37–45% in those in their 70 s [2–4]. Our cohort had a mean age of 61.7 years and a VCF incidence of 20%, which is comparable to or slightly higher than the general population. Based on these findings, early osteoporosis treatment is recommended in THA patients at risk of VCF.

Preexisting VCF and the FRAX score were identified as risk factors for the development of incident VCF. Although multivariate analysis revealed only the FRAX score as an independent predictor, this composite index incorporates several clinical parameters—including age, body composition, fracture history, corticosteroid use, and rheumatoid arthritis—supporting its utility in screening for VCF risk in patients undergoing THA.

While BMD and preexisting VCF have been established as conventional risk factors [22, 23], our study found no significant difference in lumbar BMD between hips with and without incident VCF. Several factors may account for this. First, many THA patients exhibit degenerative lumbar changes due to hip–spine syndrome, which can lead to overestimation of BMD by DEXA. Moreover, the presence of VCF itself may alter local bone morphology and density, potentially resulting in falsely high or low BMD values and thereby reducing the reliability of lumbar measurements [24]. Additionally, approximately 80% of the present cohort had osteoarthritis, a condition often associated with relatively high BMD despite increased fracture risk [25, 26]. These findings suggest that BMD alone may underestimate fracture risk in this population.

VCFs commonly occur at the thoracolumbar junction (T11–L5) [27], and in our cohort, T12 was the most frequently affected site. Since DEXA typically evaluates L1–L4 or L2–L4, this mismatch between measurement and fracture-prone sites may reduce the clinical sensitivity of BMD assessments. Therefore, preoperative osteoporosis screening should combine BMD with adjunctive tools such as the FRAX score.

Preexisting VCF was significantly associated with incident VCF (*P* = 0.002), consistent with prior studies [28]. However, neither preoperative nor postoperative osteoporosis treatment was significantly associated with reduced VCF incidence. This may reflect several confounders, including the low treatment initiation rate, limited use of osteoanabolic agents, and potential underdiagnosis or undertreatment of osteoporosis (as 35% of patients had T-scores ≤  −2.5 but only 19% received treatment). For instance, although 35% of hips had T-scores ≤  −2.5, only 19% received osteoporosis treatment preoperatively, and osteoanabolic agents were rarely used.

Notably, osteoanabolic therapies have been shown to significantly reduce VCF risk in high-risk populations and are particularly recommended for such cases. These findings highlight the need for more proactive and individualized osteoporosis management, including the potential use of osteoanabolic agents, in THA patients with elevated FRAX scores.

Implant strategy implications: Patients with high FRAX scores or preexisting VCFs may require special consideration in implant selection. VCFs are associated with increased lumbar kyphosis and pelvic retroversion, which may increase the risk of anterior dislocation after THA [7, 8, 16]. Therefore, in such patients, the use of dual-mobility cups or large femoral heads may help reduce the risk of instability. In addition, preoperative assessment of spinopelvic parameters, including pelvic tilt (PT) and lumbar lordosis (LL) angles, may be important to consider in surgical planning, as these parameters could influence the functional orientation and stability of the acetabular component. Previous studies have suggested that implant placement with consideration of pelvic tilt may improve postoperative efficacy and stability [29].

This study has several limitations. First, it is a retrospective study with a limited sample size, which warrants cautious interpretation. However, more than 80% of patients were followed for over five years, providing meaningful long-term data. Second, the study did not evaluate osteoporosis treatment adherence. Third, spinal flexibility classification (e.g., flexible vs. stiff pelvis) was not assessed, which may influence the relationship between VCF and postoperative alignment. Future prospective interventional studies are needed to determine whether pharmacological interventions—especially osteogenic agents—can reduce the incidence of VCF in patients with high FRAX scores after THA.

## Conclusions

Over an average follow-up period of 6.1 years after THA, 20% of hips developed incident VCFs, which led to postural changes and impaired hip function. The FRAX score was shown to be a useful tool for predicting the risk of VCF in patients after THA.

## Data Availability

The datasets used and/or analysed during the current study are available from the corresponding author on reasonable request.

## Abbreviations

AUC: Area under the curve
ALP: Alkaline phosphatase
BMD: Bone mineral density
BMI: Body mass index
DEXA: Dual-energy X-ray absorptiometry
FRAX: Fracture Risk Assessment Tool
HHS: Harris Hip Score
THA: Total hip arthroplasty
ROC: Receiver operating characteristic
VCF: Vertebral compression fracture
